# Insight into the Architecture of the NuRD Complex: STRUCTURE OF THE RbAp48-MTA1 SUBCOMPLEX*

**DOI:** 10.1074/jbc.M114.558940

**Published:** 2014-06-11

**Authors:** Saad S. M. Alqarni, Andal Murthy, Wei Zhang, Marcin R. Przewloka, Ana P. G. Silva, Aleksandra A. Watson, Sara Lejon, Xue Y. Pei, Arne H. Smits, Susan L. Kloet, Hongxin Wang, Nicholas E. Shepherd, Philippa H. Stokes, Gerd A. Blobel, Michiel Vermeulen, David M. Glover, Joel P. Mackay, Ernest D. Laue

**Affiliations:** From the ‡ School of Molecular Bioscience, University of Sydney, New South Wales 2006, Australia,; § Department of Biochemistry, University of Cambridge, Cambridge CB2 1GA, United Kingdom,; ¶ Department of Genetics, University of Cambridge, CB2 3EH, United Kingdom,; ‖ Department of Molecular Cancer Research, UMC Utrecht, Universiteitsweg 100, 3584CG Utrecht, The Netherlands, and; ** Children's Hospital of Philadelphia, Philadelphia, Pennsylvania 19104

**Keywords:** Chromatin, Chromatin Structure, Gene Regulation, Protein Assembly, Protein Structure, MTA1, NuRD Complex, RBBP4, RbAp48

## Abstract

The nucleosome remodeling and deacetylase (NuRD) complex is a widely conserved transcriptional co-regulator that harbors both nucleosome remodeling and histone deacetylase activities. It plays a critical role in the early stages of ES cell differentiation and the reprogramming of somatic to induced pluripotent stem cells. Abnormalities in several NuRD proteins are associated with cancer and aging. We have investigated the architecture of NuRD by determining the structure of a subcomplex comprising RbAp48 and MTA1. Surprisingly, RbAp48 recognizes MTA1 using the same site that it uses to bind histone H4, showing that assembly into NuRD modulates RbAp46/48 interactions with histones. Taken together with other results, our data show that the MTA proteins act as scaffolds for NuRD complex assembly. We further show that the RbAp48-MTA1 interaction is essential for the *in vivo* integration of RbAp46/48 into the NuRD complex.

## Teaser

**Background:** The NuRD complex controls gene expression through altering chromatin structure.

**Results:** The MTA1-RbAp48 structure shows how the RbAp46/p48 histone chaperones are recruited to NuRD.

**Conclusion:** The MTA subunits act as scaffolds for NuRD complex assembly.

**Significance:** The MTA/RbAp48 interaction prevents binding of histone H4, which is crucial for understanding the role of the RbAp46/p48 chaperones in the complex.

## Introduction

In eukaryotes, chromatin structure is modulated by multiprotein enzymes such as the nucleosome remodeling and deacetylase (NuRD) complex.^8^ NuRD can both activate and repress transcription (1–3), and it is essential for embryonic development in complex organisms (4). It also plays a role in DNA damage repair (5), and the silencing of NuRD components leads to changes in chromatin structure that mimic aging (6).

In mammals, the NuRD complex consists of ∼10 proteins that are consistently observed during purification. CHD4 (Mi-2β) is an ATP-dependent helicase that can reposition nucleosomes (7). HDAC1 and HDAC2 remove acetyl groups from lysine residues and are associated with gene repression (8). GATAD2A and GATAD2B (p66α/p66β) are potent transcriptional repressors (9, 10) but are otherwise poorly characterized, whereas MBD2 and MBD3, which appear to be mutually exclusive in NuRD, contain methyl CpG binding domains (11–13). MBD3 has been shown to play a key role in suppressing pluripotency in early development (14, 15).

RbAp46 and RbAp48 (RBBP7/RBBP4) are ∼50-kDa WD-repeat proteins (16, 17) that were first recognized for their ability to bind the tumor suppressor Rb (18). A role for RbAp48 as a mediator of age-related memory loss has also recently been reported (19). RbAp46 and RbAp48 together with HDAC1 and HDAC2 have been proposed to form a core deacetylase complex (20) that is common to both the NuRD and Sin3 complexes. RbAp46 and RbAp48 are also found in other gene regulatory complexes, such as NURF (nucleosome remodeling factor) (21) and PRC2 (polycomb repressive complex 2) (22).

**FIGURE 1.**
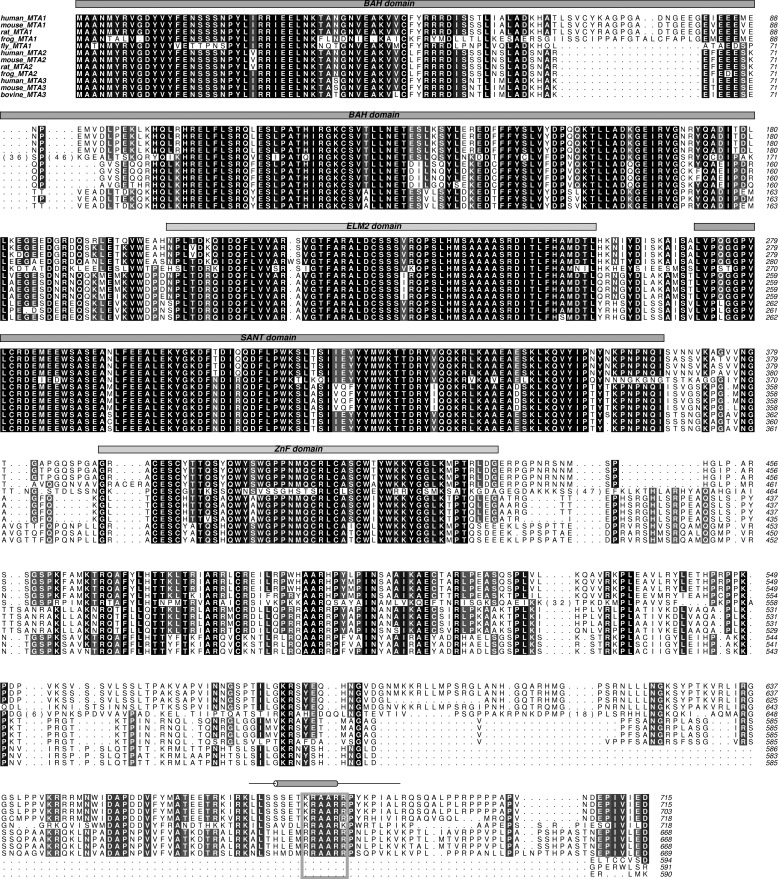
**Sequence alignment of MTA1, MTA2, and MTA3 from various eukaryotes.** The alignment was produced using the ALINE program (64). Well defined domains are indicated. Uniprot accession numbers are: human MTA1 (Q13330), mouse MTA1 (Q8K4B0), rat MTA1 (Q62599), frog MTA1 (F6YJW8), fly MTA1 (Q9VNF6), human MTA2 (O94776), mouse MTA2 (Q9R190), rat MTA2 (B2GV01), frog MTA2 (Q4V7T0), human MTA3 (Q9BTC8), mouse MTA3 (Q924K8) and bovine MTA3 (A6QL72). The KRAARR motif is *boxed in gray*.

MTA1, MTA2, and MTA3 are highly related proteins that contain several well conserved structured domains at their N termini (Fig. 1). Their C-terminal regions are less conserved and are predicted to be largely disordered. MTA1 is one of the most up-regulated genes in human cancers and is particularly associated with metastatic and aggressive tumors and with poor prognosis (23–25).

Despite its central importance in development, very little is known about the architecture of the NuRD complex, how it assembles, and which combinations of the components form stable complexes. There are three-dimensional structures available for two intact subunits, namely RbAp46/48 (16, 17) and HDAC2/HDAC1 (26, 27). There are also structures of isolated domains, including the PHD domains (28) and one chromodomain (PDB code 2EE1) of CHD4, the methyl-DNA binding domain of MBD2 (29) and the SANT domain of MTA3 (PDB code 2CRG). Although many intersubunit interactions have been reported (30), these data are almost exclusively limited to GST pulldown and co-immunoprecipitation assays. However, there are structures of the ∼40-residue coiled-coil formed between MBD2 and GATAD2A (31) as well as, notably, of the ELM2-SANT domain of MTA1 bound to HDAC1 (27). Here we show that the MTA proteins also recruit the histone chaperones RbAp46/48, suggesting that MTA1/2/3 act as a scaffold in NuRD. Moreover, our structure of the MTA1-RbAp48 complex shows that the interaction of MTA subunits with RbAp46/48 modulates binding of histones H3-H4, a substrate of the complex.

## EXPERIMENTAL PROCEDURES

### 

#### 

##### Plasmid Construction

FLAG-tagged human RbAp48 (Uniprot: Q09028) was constructed by PCR and cloned into pcDNA3. MTA1 constructs (residues 1–250, 230–550, and 530–715) were generated by PCR amplification of the human gene (Uniprot: Q13330) and cloned into pcDNA3. MTA1 constructs (residues 530–635, 625–715, 643–695, and 656–686) were created by PCR amplification from codon-optimized sequences for bacterial expression (GenScript) and cloned into pGEX-6P with an N-terminal GST tag.

##### Protein Production for Biochemical/Structural Analysis

pcDNA3 constructs were expressed *in vitro* in the presence of [^35^S]methionine. GST-MTA1 constructs were expressed in the BL21 strain of *Escherichia coli* and induced with 0.4 mm isopropyl 1-thio-β-d-galactopyranoside. The culture was incubated with shaking at 25 °C overnight, and the protein was subsequently purified by glutathione affinity chromatography. GST-H4-(1–48) was expressed and purified by glutathione affinity chromatography as described previously (16). Recombinant RbAp48 was expressed and purified from insect cells as described previously (17).

##### Peptide Synthesis

All MTA1 peptides used here were synthesized and purified (to 95% purity) by ChinaPeptides (Shanghai, China) with acetyl- and amide-capping groups at the N- and C-terminal ends, respectively.

##### Pulldown Assays

RbAp48 and MTA1 constructs were *in vitro* translated in the presence of ^35^S-labeled methionine using the TNT Quick Coupled Transcription/Translation kit (Promega) according to the manufacturer's instructions. *In vitro* translated ^35^S-labeled FLAG-RbAp48 (20 μl) was immobilized onto agarose beads conjugated to anti-FLAG (M2) antibodies (Sigma) in binding buffer (50 mm Tris, pH 7.5, 300 mm NaCl, 0.5% Triton X-100, complete protease inhibitor mixture tablet (Roche Applied Science), and 1 mm DTT). The mixture was incubated with *in vitro* translated ^35^S-labeled-MTA1 fragments (40 μl) at 4 °C for 2 h. The beads were then washed five times with binding buffer. Anti-FLAG beads alone were used as a negative control. Complexes were resolved by SDS-PAGE and visualized by autoradiography.

GST pulldown assays were performed by incubating equal amounts of GST or GST-MTA1 fusion proteins immobilized on glutathione-Sepharose beads (Amersham Biosciences) with *in vitro* translated ^35^S-labeled RbAp48. The mixtures were incubated for 2 h at 4 °C and washed 5 times with binding buffer. Complexes were resolved by SDS-PAGE and visualized by autoradiography.

##### Isothermal Titration Calorimetry (ITC)

ITC measurements were carried out at 25 °C using a MicroCal iTC200 titration calorimeter. RbAp48 and MTA1 peptides were dialyzed separately overnight against a buffer containing 20 mm Tris, pH 7.5, and 150 mm NaCl. The MTA1 peptide (250 μm) was titrated into RbAp48 (25 μm, 200 μl) in a series of 20 (2 μl) injections with a 2.5-min interval between each injection. The reference power was set to 2 μcal/s, and the cell was stirred continuously at 1000 rpm. The evolved heats were integrated and normalized for protein concentration. After base-line correction (using data from the titration of the MTA1 peptide into buffer), the data were fitted to a simple single-site binding model using the MicroCal Origin 7.0 software package.

##### Crystallization and Structure Determination

MTA1 peptides (comprising residues 656–686, 670–695, and 670–711) were dissolved in 50 mm Tris, pH 8.0. Concentrated RbAp48 protein (5 mg/ml) was mixed with MTA1 peptide at a molar ratio of 1:4. Co-crystallization trials were set up in 96-well plates as sitting drops using either an Oryx6 protein crystallization robot (Douglas Instruments) or Nanodrop Mosquito robot and commercial crystallization screens (Morpheus (Gorrec 2009) and NeXtalDWBlocks, Qiagen). Crystals grew in 1–2 days at 18 °C in several conditions. X-ray data were collected for the RbAp48-MTA1-(656–686) complex from single crystals obtained in 10% w/v polyethylene glycol (PEG) 20,000, 20% v/v polyethylene glycol monomethyl ether 550, 0.2 m 1,6-hexanediol, 0.2 m 1-butanol, 0.2 m (*RS*)-1,2-propanediol, 0.2 m 2-propanol, 0.2 m 1,4-butanediol, 0.2 m 1,3-propanediol, and 0.1 m MES/imidazole, pH 6.5. For the RbAp48-MTA1-(670–695) complex, data were collected from crystals obtained in 12.5% w/v PEG 1000, 12.5% w/v PEG 3350, 12.5% v/v 2-methyl-2,4-pentanediol, 0.2 m 1,6-hexanediol, 0.2 m 1-butanol, 0.2 m (*RS*)-1,2-propanediol, 0.2 m 2-propanol, 0.2 m 1,4-butanediol, 0.2 m 1,3-propanediol, and 0.1 m MES/imidazole pH 6.5. Crystals of RbAp48-MTA1-(670–711) grew in 0.2 m calcium acetate and 20% w/v PEG 3350. Crystals were harvested in mother liquor before cryogenic cooling in liquid nitrogen. All crystals were cryo-protected with the addition of 25% glycerol. Diffraction data were collected at the Diamond Light Source beamline I04 (for crystals of RbAp48-MTA1-(656–686)/(670–695)) and using an in-house rotating copper anode generator (for crystals of RbAp48-MTA1-(670–711)). Data were processed and scaled using MOSFLM (32) and Scala (33). The structures were solved by molecular replacement with the program Phaser (34) using RbAp48 as the search model (PDB code 2XU7). Models were built with Coot (35) and refined using Refmac (36). MolProbity (37) was used to validate each structure, and the EBI PISA server (38) was used for interface analysis.

The atomic coordinates and structure factors for the complexes of RbAp48 bound to MTA1-(656–686), MTA1-(670–695) and MTA1-(670–711) have been deposited into the Protein Data Bank (PDB entries 4PBY, 4PBZ, and 4PC0, respectively).

##### Expression and Affinity Purification of Protein A- and GFP-tagged Complexes from Drosophila melanogaster Cells

Full-length Nurf55 (also known as p55 and CAF1) is the *D. melanogaster* homolog of RbAp46/p48. It was PCR-amplified and cloned into the pDONR221 vector via the BP (recombination and insertion of the att B sequence into the att P recombination site) reaction using standard Gateway Technology (Invitrogen) protocols. Five residues in this plasmid were mutated (L35Y, E361Q, D362N, E364Q, D365N) to create pDONR-Nurf55_mut_ using the QuikChange Lightning Site-directed Mutagenesis kit (Agilent Technologies). The resulting wild-type and mutant clones were verified by sequencing. Primer sequences for the constructs are available on request. The Nurf55 genes were then transferred into destination vectors for the inducible expression of protein A- or GFP-tagged fusion proteins by the LR (insertion of sequences containing att *L* sites into destination att *R* sites) reaction according to the manufacturer's instructions.

*D. melanogaster* Dmel-2 cell lines stably expressing either tagged wild-type or mutant Nurf55 (containing the L35Y, E361Q, D362N, E364Q, and D365N mutations) were established. Affinity purification of protein A- and GFP-tagged bait and interacting proteins followed by mass spectrometry identification was carried out as previously reported (39–41).

## RESULTS

### 

#### 

##### A Short Motif at the C-terminal End of MTA-1 Binds RbAp48

**FIGURE 2.**
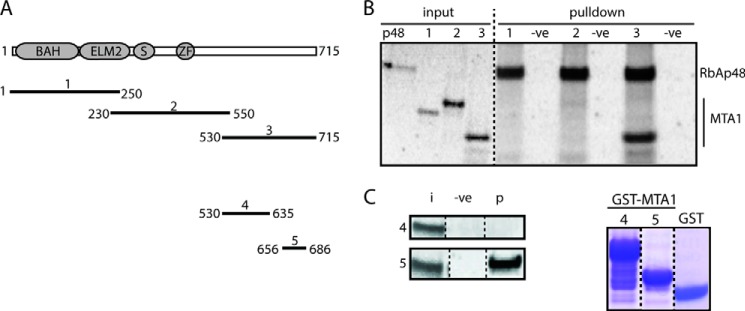
**Mapping the region of MTA1 that binds RbAp48.** *A*, schematic of the MTA1 constructs used in pulldown assays. *B*, pulldown assays using *in vitro* translated ^35^S-labeled FLAG-RbAp48 immobilized on anti-FLAG beads and *in vitro* translated ^35^S-labeled MTA1 constructs. Input is 10% of the amount used in each pulldown. A sample containing beads alone served as a negative control. Reactions were analyzed by SDS-PAGE and autoradiography. *C*, *left hand panel*, pulldown assays using *in vitro* translated ^35^S-labeled RbAp48 pulled down by bacterially expressed GST fusions of MTA1 fragments loaded onto glutathione-Sepharose beads. Input (*i*) refers to 10% of the amount of RbAp48 used in each pulldown, and the negative control (−*ve*) contains GST alone loaded onto beads. Protein (*p*) refers to RbAp48 protein that is pulled down in each case. *Right hand panel*: Coomassie blue-stained SDS-PAGE indicating the amount of either GST or GST-MTA1 (constructs 4 and 5) used.

To delineate the RbAp48 binding region of MTA1, we generated a series of *in vitro* translated N- and C-terminal truncations of MTA1 and carried out pulldown experiments using *in vitro* translated FLAG-RbAp48 as bait. N-terminal constructs encompassing the BAH, ELM2, SANT, and ZF domains of MTA1 did not interact with RbAp48 (Figs. 2, *A* and *B*), whereas the C-terminal portion of MTA1 was robustly pulled down. Pulldown experiments using additional deletion constructs (Fig. 2*C*) indicated that MTA1-(656–686) was sufficient to bind RbAp48. Comparison of this sequence with those of other RbAp48/RbAp46-binding proteins revealed a short motif (^678^KRAARR^683^) that resembles part of the RbAp48/RbAp46 binding motif of histone H4 (16, 42). In histone H4, this motif adopts a helical conformation when bound to RbAp46. As MTA1(∼665–682) was also predicted to be helical, we wondered whether it might have a similar binding mode.

##### Crystal Structures of RbAp48 Bound to MTA1 Peptides

**FIGURE 3.**
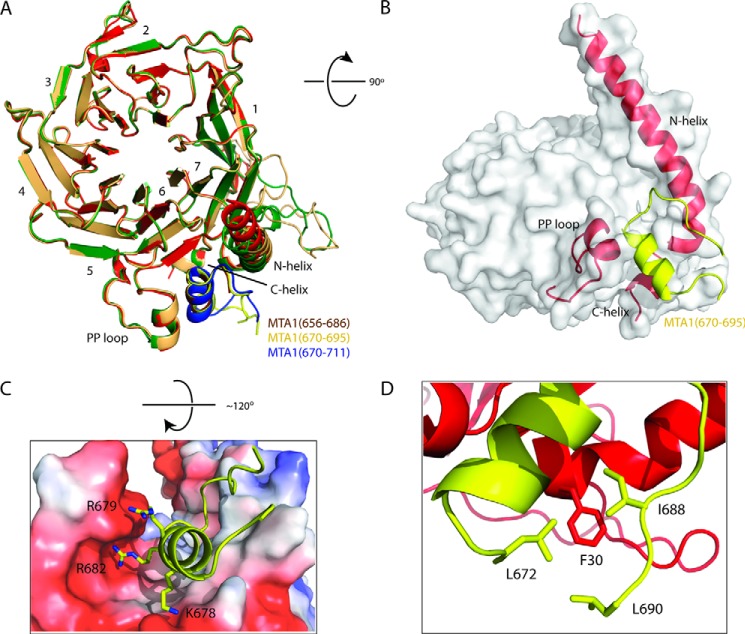
**X-ray crystal structures of RbAp48-MTA1-(656–686), RbAp48-MTA1-(670–695), and RbAp48-MTA1-(670–711).** *A*, ribbon diagram showing a superposition of RbAp48-MTA1-(656–686), RbAp48-MTA1-(670–695), and RbAp48-MTA1-(670–711) over all Cα atoms. RbAp48 is shown in *green*, *red*, and *gold*, and the MTA1 peptides are shown in *brown*, *yellow*, and *blue*, respectively. The blades of RbAp48 are labeled numerically. *B*, surface/schematic view of RbAp48-MTA1-(670–695), indicating the elements of the RbAp48 structure that are contacted by MTA1. The structure is rotated 90° in the indicated direction relative to the orientation in part A. *C*, detail of the RbAp48-MTA1-(670–695) structure showing interactions made by the basic side chains of MTA1. Here, the structure is rotated 120° relative to its orientation in part A. *D*, hydrophobic interactions formed in the complex. Residues from RbAp48 are shown as *red sticks*, and MTA1 is shown in *yellow*.

The shortest fragment of MTA1 capable of pulling down *in vitro* translated RbAp48 was MTA1-(656–686) (Fig. 2), and so to understand the mode of binding we crystallized a complex of RbAp48 bound to MTA1-(656–686) (Fig. 3). Diffraction data were collected to 2.50 Å resolution and indexed in the space group P2_1_. Phases were obtained by molecular replacement using the structure of RbAp48 (PDB code 2XU7) as a search model (17). MTA1 was clearly detectable in the histone H4 binding site on RbAp48 in an m*F_o_* − D*F_c_* difference map. The KRAARR motif of the MTA1 peptide was observed, but residues N-terminal to this sequence (656–672) did not give clear electron density, suggesting that they were not ordered. We, therefore crystallized two further RbAp48-MTA1 complexes, where the N terminus of each MTA1 peptide was defined by the structurally resolved region in our initial complex (residue 670). We extended the C terminus of each MTA peptide (either to residue 695 or 711) to explore the extent of the structured segment and collected diffraction data to 2.15 Å and 2.50 Å resolution, respectively. Two molecules of RbAp48 and two molecules of MTA1 were found in the asymmetric unit of crystals formed from the complexes of RbAp48 and MTA1-(656–686 and 670–711), whereas one molecule of each polypeptide was observed in the asymmetric unit of RbAp48-MTA1-(670–695). *R*_cryst_ and *R*_free_ for each of the final refined models were, respectively, 18.2% and 22.5% for RbAp48-MTA1-(656–686), 18.9% and 23.1% for RbAp48-MTA1-(670–695), and 20.6% and 25.9% for RbAp48-MTA1-(670–711). The statistics for data collection and refinement are listed in Table 1.

**TABLE 1:** **Crystallographic and refinement statistics for the RbAp48/MTA1 complexes** r.m.s.d., root mean square deviation; I, intensity; ChirVolume, chiral volume.

	RbAp48-MTA1-(656–686)	RbAp48-MTA1-(670–695)	RbAp48-MTA1-(670–711)
**Data Collection**			
Space group	P2_1_	P2_1_	P2_1_
Unit cell dimensions			
*a*, *b*, *c* (Å)	81.56, 59.48, 104.63	61.80, 59.82, 68.07	52.25, 123.23, 87.34
α, β, γ (°)	90.00, 90.12, 90.00	90.00, 99.19, 90.00	90.00, 103.39, 90.00
*R*_esolution_ (Å)	52.31-2.50 (2.64-2.50)	33.60-2.15 (2.21-2.15)	61.61-2.50 (2.64-2.50)
*R*_merge_ (%)	12.2 (44.8)	4.7 (49.7)	9.50 (35.7)
No. of observations	122,920 (18,058)	94,637 (4,598)	234,305 (33,787)
Mean [(*I*)/S.D. (*I*)]	10.6 (2.4)	13.0 (2.1)	11.9 (3.8)
Completeness (%)	99.6 (99.8)	97.3 (80.8)	91.1 (100.0)
Multiplicity	3.5 (3.6)	3.6 (2.9)	6.9 (6.3)

**Refinement**			
Resolution (Å)	20.00-2.50	20.00-2.15	20.00-2.50
No. of reflections	33,113	24,747	32,008
Rwork/Rfree	0.182/0.225	0.189/0.231	0.206/0.259
r.m.s.d. values			
Bond length (Å)	0.011	0.013	0.006
Bond angles (°)	1,432	1,494	1,041
ChirVolume	0.082	0.087	0.061
No of atoms	6,768	3,378	7,094
B-factor	25.49	61.13	33.57
Ramachandran values (%)			
Most favored	95.0	95.8	97.0
Additionally allowed	4.5	3.9	2.8
Outliers	0.5	0.3	0.2

**FIGURE 4.**
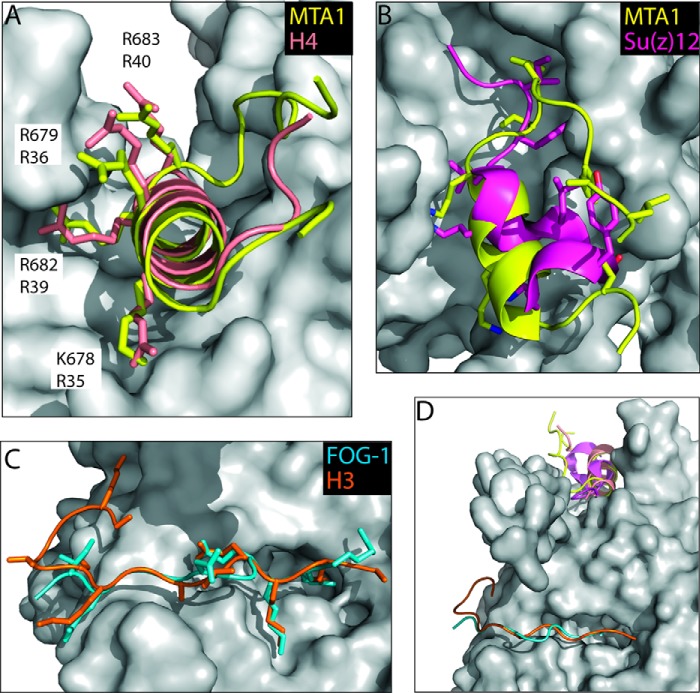
**Comparison of the RbAp48-MTA1 structure with other RbAp46/48 complex structures.** *A*, comparison of interactions made by histone H4-(28–42) (PDB 3CFS, *salmon*) and MTA1-(670–695) (*yellow*) with RbAp46 (16) and RbAp48, respectively. Residues in MTA1 and H4 that make intermolecular contacts are shown as *sticks*. All of the interactions made by histone H4 are observed in the MTA1 structure. Key interacting residues of MTA1 together with the corresponding H4 residues are labeled. *B*, comparison of interactions made by Su(z)12-(79–91) (PDB 2YB8, *magenta*; Ref. 43) and MTA1-(670–695) (*yellow*) with Nurf55 and RbAp48, respectively. Residues in MTA1 and Su(z)12 that make intermolecular contacts are shown as *sticks. C*, comparison of interactions made by H3-(2–20) (PDB code 2YB8, *orange*; Ref. 43) and FOG-1-(1–15) (PDB code 2XU7, *cyan*; Ref. 17) with Nurf55 and RbAp48, respectively. *D*, overlay of RbAp46/48/Nurf55-MTA1/H4/Su(z)12/H3/FOG-1 complex structures. In all parts, RbAp48 is shown as a *gray surface*.

The conformations of RbAp48 were essentially identical across all three structures (root mean square deviation of 0.21 Å pairwise over 311 Cα atoms; Fig. 3). RbAp48 has the seven-bladed β-propeller fold common to all WD40-repeat proteins but also displays three non-canonical features: an extended loop (the PP loop; Ref. 16) inserted into blade 6, a long additional N-terminal α-helix that contacts blades 6 and 7, and a single turn of C-terminal helix after blade 7. Our structures reveal that the MTA1 peptide binds in an amphipathic α-helical conformation in the groove on RbAp48 formed by these three structural elements (Fig. 3). As predicted by its sequence similarity with histone H4, the basic residues in the MTA1 KRAARR motif make very similar hydrogen bonding and salt bridge contacts with this acidic groove (Fig. 3*C*). Residues Lys-678/Arg-682 and Arg-679/Arg-683 of MTA1 make analogous contacts to those formed with residues Arg-35/Arg-39 and Arg-36/Arg-40 of histone H4, respectively (Fig. 4*A*). The presence of electron density from the MTA1 sequence KRAARR in all three structures strongly suggests that this motif is critical for the interaction between RbAp48 and MTA1.

##### MTA1-(670–695) Has a Higher Affinity for RbAp48 Than Does MTA1-(656–686) or MTA1-(670–711)

**FIGURE 5.**
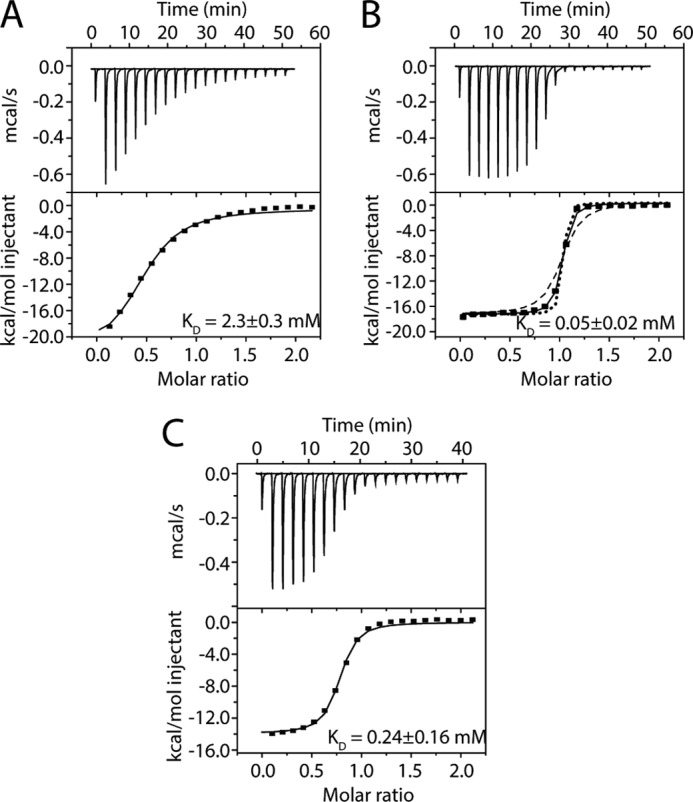
**Measurement of the binding affinities of MTA1 peptides for RbAp48 by ITC.** *A*, ITC profile for binding of an MTA1-(656–686) peptide to RbAp48. *B*, ITC profile for binding of an MTA1-(670–695) peptide to RbAp48. Simulated curves for affinities of 0.016 and 0.15 μm are shown to provide an indication of the reliability of the fit. *C*, ITC profile for binding of an MTA1-(670–711) peptide to RbAp48. In all cases data were fitted to a one-site model with a stoichiometry of 1:1. Titrations were carried out at 25 °C, and the data were fitted using Origin 7.0 and a standard 1:1 binding model (*solid black line in the lower panel*). Each titration was carried out in duplicate.

As expected, pulldown experiments with point mutants confirmed that the KRAARR motif is essential for RbAp48 recognition by MTA1 (data not shown). We also confirmed the importance of this motif using isothermal titration calorimetry. In all cases the data fitted well to a simple 1:1 binding isotherm, and dissociation constants from representative measurements were 2.3 ± 0.3, 0.05 ± 0.007, and 0.24 ± 0.16 μm for MTA1-(656–686), MTA1-(670–695), and MTA1-(670–711), respectively (Fig. 5; standard errors from single curve fits are given). The structures provide a rationale for the higher affinity observed for the MTA1-(670–695) and MTA1-(670–711) peptides as compared with MTA1-(656–686). As shown in Fig. 3*D*, residues Ile-688 and Leu-690 form a hydrophobic cluster with Leu-672. This cluster, which cannot form in the MTA1-(656–686) peptide, interacts with the aromatic side chain of Phe-30 in RbAp48. It is not clear at this stage, however, why the affinity of RbAp48 for the MTA1-(670–711) peptide is apparently lower than that for MTA1-(670–695); the interactions made in both crystal structures are essentially identical. Our data show that the extreme C terminus of the protein (Leu-696–Asp-715) is not required for the interaction.

##### MTA1 and Histone H3/FOG1 Bind Independently to RbAp48

**FIGURE 6.**
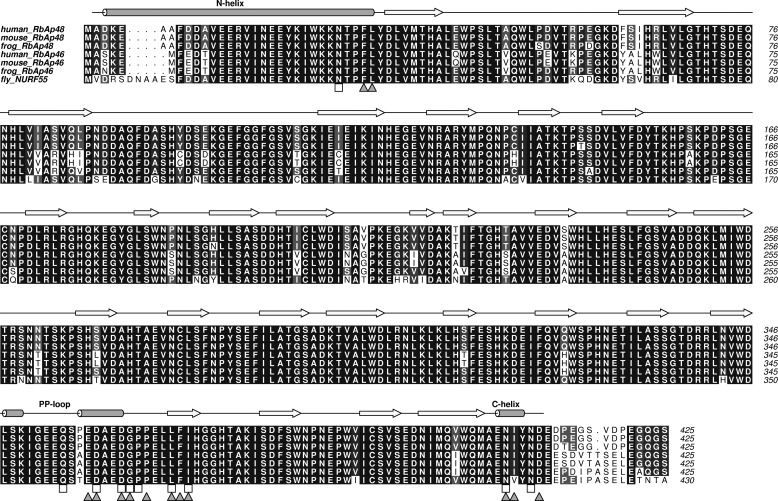
**Sequence alignment of the WD40 proteins RbAp48 and RbAp46 from *Homo sapiens*, *Mus musculus,* and *Xenopus tropicalis* with *D. melanogaster* (Nurf55).** Secondary structure (with reference to RbAp48) is denoted by *arrows* (β-strands) and *cylinders* (α-helices), and the MTA1 (*squares*) and H4 binding residues (*triangles*) are indicated.

**FIGURE 7.**
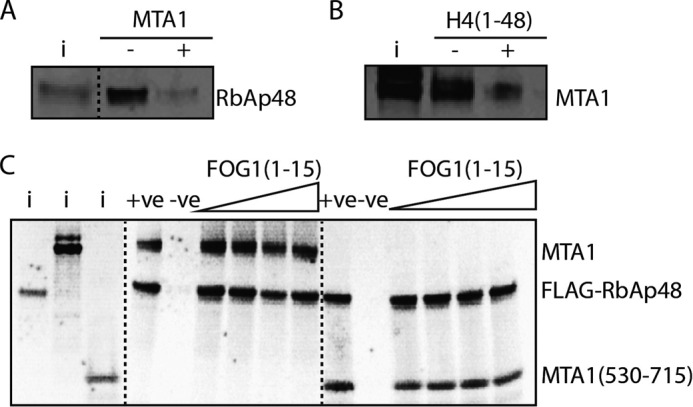
**Histone H3/FOG1 and MTA1 do not compete for binding to RbAp48.** *A,* pulldown showing the binding of *in vitro* translated ^35^S-labeled RbAp48 to GST-histone H4-(1–48) (immobilized on glutathione beads) in the absence and presence of *in vitro* translated MTA1. *B,* pulldown showing the binding of *in vitro* translated ^35^S-labeled MTA1 to FLAG-RbAp48 (immobilized on anti-FLAG beads) in the absence and presence of histone H4-(1–48). *C,* pulldown assays showing the effect of adding increasing amounts of FOG1-(1–15) peptide to a complex formed between *in vitro* translated ^35^S-labeled FLAG-RbAp48 (immobilized on anti-FLAG beads) and *in vitro* translated ^35^S-labeled full-length MTA1 or MTA1-(530–715). The input (*i*) lane contains 10% of the ^35^S-labeled RbAp48, full-length MTA1, and MTA1-(530–715) proteins used in pulldown assays. The positive control (+*ve*) is ^35^S-labeled FLAG-RbAp48 and MTA1 without the FOG1-(1–15) peptide, and anti-FLAG beads plus MTA1 alone was used as the negative control (−*ve*).

The structural data provide clear evidence that histone H4 and MTA1 bind mutually exclusively to RbAp48. We previously solved the structure of a complex formed by RbAp48 and a peptide (residues 1–15) derived from the co-regulator FOG1 (17), demonstrating that the peptide binds in an extended conformation to a groove on one face of the RbAp48 β-propeller (Fig. 4*C*). Schmitges *et al.* (43) showed that the N-terminal tail of histone H3 binds to Nurf55 in a very similar fashion to FOG1 (Fig. 4*C*) (Nurf55, also known as p55 and CAF1, is the sole *D. melanogaster* homolog of RbAp46/RbAp48; see Fig. 6). Competition-binding experiments were performed to assess whether full-length MTA1 might inhibit the interaction of RbAp48 with H3 or FOG1 peptides and to confirm that H4 and MTA1 bind mutually exclusively to RbAp48. GST-H4-(1–48) was expressed in bacteria, immobilized onto glutathione beads, and used to pull down RbAp48 in the absence and presence of full-length MTA1 (Fig. 7*A*). In a complementary experiment (Fig. 7*B*), *in vitro* translated FLAG-RbAp48 was immobilized on anti-FLAG beads and used to pull down MTA1 in the absence and presence of histone H4-(1–48). These data support the conclusion that histone H4 and MTA1 compete for the same binding surface on RbAp48. To examine whether MTA1 can also inhibit interactions with either histone H3 or FOG1, a competition experiment was performed by adding increasing amounts of FOG1-(1–15) peptide to a complex comprising full-length MTA1 and FLAG-RbAp48 immobilized on anti-FLAG beads (Fig. 7). In this experiment the FOG1 peptide was unable to interfere with the MTA1-RbAp48 interaction. The same result was observed using only a C-terminal MTA1 fragment (residues 530–715) that includes the KRAARR motif identified above. Thus, it is likely that the interaction of MTA1 with RbAp48 does not affect the interactions with either histone H3 or FOG1.

##### The KRAARR Binding Motif Is Required for the Interaction of RbAp48 with NuRD in Vivo

Next, we sought to determine whether RbAp48 and MTA1 interact in the same way *in vivo*, and, if so, whether the interaction of RbAp48 with the NuRD complex is affected when the RbAp48-MTA1 interaction we have identified is abolished. To this end, we studied the simpler *D. melanogaster* NuRD in which each protein has only a single isoform. All components bear a high degree of sequence similarity to their mammalian counterparts, rendering this a good model for the mammalian NuRD complex.

**FIGURE 8.**
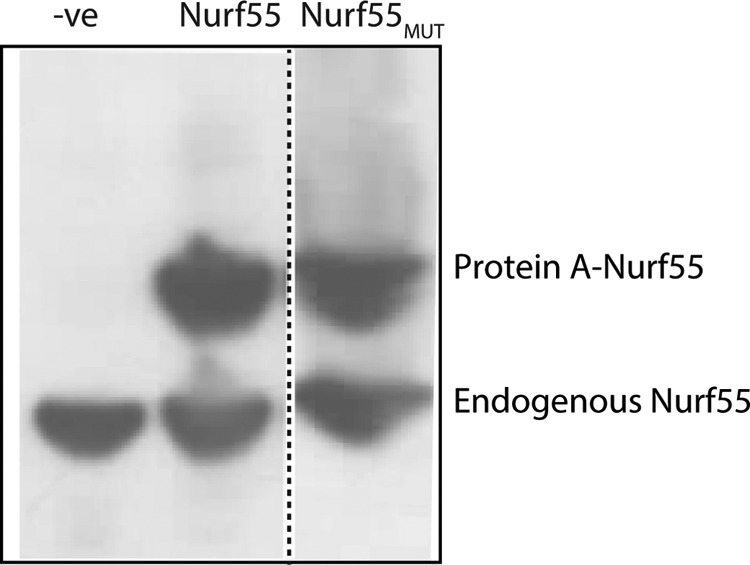
**A comparison of endogenous and protein A-tagged Nurf55.** Western blot analysis to compare the expression levels of stably expressed protein A-tagged and endogenous Nurf55 in *D. melanogaster* Dmel-2 cells using an anti-p55/dCAF1 (ab1766) antibody at 1:5000 dilution.

Nurf55 has previously been crystallized in complex with a peptide from histone H4, and this structure is in close agreement with that of RbAp46 bound to the same peptide (16, 44, 45). We designed a version of Nurf55 in which residues lining the MTA1/histone H4 binding pocket were mutated. Cell lines stably expressing protein A-tagged fusion proteins of either wild-type Nurf55 or the mutant (Nurf55_mut_) were generated. RbAp46 shares >90 and 85% sequence identity with RbAp48 and NuRF55, respectively. We previously demonstrated that when RbAp46 is mutated within its charged PP loop (E356Q, D357N, E359Q, and D360N) and within the hydrophobic surface of helix 1 (L30Y), these collective mutations abrogate its interaction with histone GST-tagged H4 (residues 1–48; Ref 16). We, therefore, made analogous changes to the Nurf55 binding pocket (E361Q, D362N, E364Q, D365N, and L35Y) to similarly disrupt the interaction of Nurf55 with H4/MTA1. GFP-tagged versions of these proteins were used to confirm that they share the same subcellular localization as the endogenous protein (data not shown). We also confirmed that the Nurf55/Nurf55_mut_ fusion proteins were expressed at endogenous levels (Fig. 8). Cellular proteins that interact with protein A-tagged Nurf55/Nurf55_mut_ were then isolated by affinity purification on IgG beads and identified by mass spectrometry as described previously (39, 40).

All of the known subunits of the *D. melanogaster* NuRD complex (MTA-like, MBD-like, Rpd3 (dHDAC), and Mi-2 (dCHD)), were pulled down by Nurf55, whereas the equivalent interactions with Nurf55_mut_ were not observed (see Table 2). We also identified Simjang and CG18292 as possible *Drosophila* homologs of the mammalian GATAD2A/2B (p66α/p66β) and CDK2AP (DOC1 (46)) proteins, respectively. To confirm these results we carried out a second experiment in which cellular proteins that interact with GFP-tagged Nurf55/Nurf55_mut_ were isolated by affinity purification on beads to which a recombinant anti-GFP nanobody had been covalently attached (41). This experiment allowed more efficient purification of the complex and thus better coverage of tryptic peptides from the Mi-2, Rpd3, Simjang, and CG18292 proteins with wild-type Nurf55. However, once again the numbers of NuRD complex peptides detected with the Nurf55 mutant were reduced greatly (see Table 2), confirming that disruption of the RbAp48-MTA1 interface we identified abolishes the association of RbAp48 with the NuRD complex. These data underscore the *in vivo* importance of the RbAp48-MTA1 interaction we identified for the association of RbAp48 with the NuRD complex.

**TABLE 2:** **Identification of Nurf55 interactors in *D. melanogaster* Dmel-2 cells** Peptides were affinity-purified using either wild type (WT) or mutated (MUT) Nurf55-protein A (top) or Nurf55-GFP (bottom) fusion proteins as bait. For the GFP affinity purification, each experiment was performed in triplicate, and the lowest numbers of unique peptides derived from NuRD subunits are listed. In the Nurf55 mutant, the following Nurf55 residues were mutated to prevent binding to either MTA1 or H4 peptides: E361Q, D362N, E364Q, D365N, and L35Y.

Protein name	Number of peptides identified	
	Nurf55 WT	Nurf55 MUT
**Protein A affinity purification**		
MTA-like	57	0
MBD-like	35	0
Simjang	9	0
dMi-2	6	0
Rpd3	8	0
CG18292 (DOC1)	2	0

**GFP affinity purification**		
MTA-like	79	6
MBD-like	22	2
Simjang	21	4
dMi-2	49	14
Rpd3	36	9
CG18292 (DOC1)	4	0

## DISCUSSION

### 

#### 

##### The RbAp48-MTA1 Interaction Is Highly Conserved

Previous studies using GST pulldown assays have mapped the domains that mediate the interaction between MTA1/2 and RbAp46/48. Roche *et al.* (47) showed that the predicted GATA-type zinc finger domain of MTA1 (residues 392–448) is sufficient to recognize RbAp46/48, and Fu *et al.* (48) reported that RbAp46 could interact with any of three non-overlapping constructs from the C-terminal portion of MTA2; only one of these constructs, however, contained the predicted zinc finger. Our crystallographic and biochemical data reveal that a short motif near the C-terminal end of MTA1 is able to bind independently to RbAp48. Specifically, residues 672–688 of MTA1 adopt a conformation that includes a short amphipathic α-helix, binding in a distinct pocket formed by three helical elements of RbAp48. Although the apparent inconsistency between these different studies remains unresolved, it is worth noting that GST pulldown assays can give rise to false positive interactions if the domain fused to GST is not correctly folded (49, 50).

Despite the apparent divergence of the C-terminal regions of the MTA proteins (*e.g.* there is 34% sequence similarity for residues 431–715 of human MTA1/2 compared with 74% for residues 1–430), inspection of the amino acid sequences of MTA2/MTA3 and RbAp46 indicates that these proteins should be able to substitute for MTA1 and RbAp48, respectively, to form analogous interactions (Figs. 1 and 6). Furthermore, a comparison of the sequences of related proteins from diverse organisms that are predicted to contain a NuRD-like complex (Figs. 1 and 6) suggests that this interaction is broadly conserved. Therefore, it is likely that the interaction we have identified represents a core element of NuRD architecture across all organisms.

##### The MTA1 Interaction Is Critical for RbAp48 Interactions with the NuRD Complex

Our mass spectrometry data probing NuRD interactions in *Drosophila* cells underscore the importance of the RbAp48-MTA1 interaction on several levels. First, the data show that the MTA1 interaction is essential for proper assembly of RbAp48 into NuRD (in the context of the full-length, endogenous versions of these proteins) and is conserved across >400 million years of evolution. Second, they corroborate the MTA1-binding site that we have identified and suggest that this binding surface is the major (if not only) site of contact between Nurf55 and the NuRD complex.

##### MTA1 and Histone H4 Compete for the Same Binding Site on RbAp48

Our data show clearly that MTA1 and histone H4 bind to the same surface of RbAp46/48. A comparison of the RbAp48-MTA1 and RbAp46-H4 structures shows that essentially all of the intermolecular interactions made by histone H4 side chains are recapitulated in the RbAp48-MTA1 complex (Fig. 4*A*). Our crystal structures suggest why the affinities of the longer MTA1 peptides for RbAp48 are substantially higher than those of histone H4 peptides for RbAp46 (*K_D_* of 0.05 μm for MTA1-(670–695) and 0.2 μm for MTA1-(670–711) *versus* 1 μm for H4-(1–48) (16)). A tyrosine at position 685 in MTA1 is positioned in a narrow groove on the surface of RbAp48, forming van der Waals contacts with residues that line the groove, but this residue is replaced with a glycine in histone H4.

As noted above, the RbAp46/48 proteins are found in a number of chromatin complexes, including SIN3 (51), PRC2 (22), NURF (21), HAT1 (42), and CAF1 (52), and our results suggest that they may play very different roles in each setting. Within the NuRD complex the results show that RbAp46/p48-H3-H4 interactions are destabilized in the presence of MTA proteins, but that RbAp46/p48 can still interact with histones H3-H4 through the N-terminal tail of histone H3 (43). In other words our results suggest that competition between MTA1 and histone H4 inhibits the capacity of RbAp46/48 (in the context of NuRD) to interact with histones H3-H4 as tightly as they would otherwise (RbAp48 binds the histone H3-H4 complex with a *K_D_* of 0.6 nm; Ref. 53).

##### Interactions between RbAp48 and Histones H3-H4 Might be Modulated in Several Chromatin Complexes

Inspection of the structure of the RbAp48-MTA1 peptide complex reveals that the binding surface on RbAp48 occupied by the MTA1 peptide and histone H4 is also shared with Su(z)12, a component of PRC2 (43). Interestingly, however, whereas a 12-residue peptide from Su(z)12 occupies the H4/MTA1-binding site of Nurf55, it does not form an α-helix (43). The Su(z)12 backbone has instead an irregular conformation, which nevertheless places several residues into positions that mimic the interactions made by MTA1 with RbAp48 (Fig. 4*B*). Thus, Su(z)12_R85_ occupies the same pocket as MTA1_R682_, and Su(z)12_L87/Y89/F82_ forms a hydrophobic cluster that interacts with Nurf55_F34_ (the equivalent of RbAp48_F30_). Similarly, as shown in Fig. 4*C* (17, 43), FOG1 and H3 make highly conserved specific interactions with RbAp48/Nurf55 (distinct from that of MTA1, histone H4, and Su(z)12). The RbAp46/RbAp48/Nurf55 proteins are, therefore, versatile interactors capable of contacting multiple partners via the same interaction sites.

The RbAp46/48 proteins, which carry no known enzymatic activity or nucleic acid binding capacity, act as chaperones for histones H3-H4 in the HAT1 (42) and CAF1 (52) complexes. The finding that both MTA1 (this work) and Su(z)12 (45) bind in the same site as histone H4 suggests that the very tight binding of the histone H3-H4 complex by the RbAp46/48 proteins may be also be modulated in PRC2 as well as in NuRD.

##### MTA Proteins Act as a Scaffold for NuRD Complex Assembly and the Interaction with Chromatin through Multiple Interfaces

**FIGURE 9.**
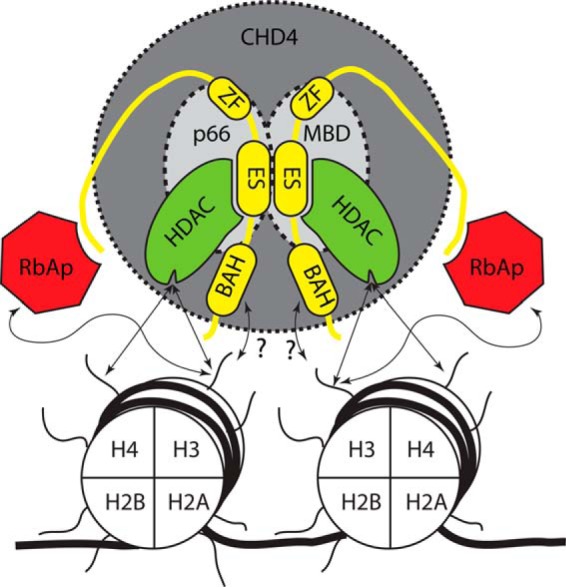
**A representation of the intracomplex and nucleosomal interactions of mammalian NuRD, facilitated by the RbAp48-MTA1 interaction.** RbAp48 (*red*) interacts with MTA1 (*yellow*, this work) and also with the histone H3 tail (*arrows*; Refs. 43 and 44). HDAC1/2 (*green*) interact with the MTA1 ELM+SANT domains (ES; Ref. 27). The BAH domain of MTA1 may also interact with nucleosomes (*arrows* + ?). HDAC1/2 remove acetyl groups on histone tails. MBD2/3 and p66a/b are shown in the background, as is CHD4 (domains from CHD4 also interact with nucleosomes but are not shown here for clarity (62, 63).

When taken together with the structure of the MTA1-HDAC1 complex (27) and recent studies of MTA1/CHD4 interactions (54), our work suggests that the MTA proteins act as a scaffold for NuRD complex assembly, bringing together multiple proteins that interact with histones, DNA, and nucleosomes (summarized in Fig. 9). The dimeric MTA1 recruits, via its ELM2-SANT domains (27), two molecules of HDAC1, which in turn can bind and deacetylate histone H3 (55). Our results suggest that the association of NuRD with histones H3-H4 might be mediated (in the absence of interactions with transcriptional co-regulators such as FOG1) by binding of RbAp46/48 to the N-terminal tail of histone H3. Consistent with this conclusion, MTA1 and MTA2 have both been reported to bind the N-terminal tail of H3 in a manner that is inhibited by methylation of H3K4 (54, 56). The BAH domains of several transcriptional regulators, including ORC1b (57) and CMT3 (58), have also been demonstrated to act as histone recognition modules. MTA1 contains an N-terminal BAH domain (Fig. 1), and this too may be involved in nucleosome interactions. The recruitment of CHD4 through MTA1 interactions also allows a number of additional contacts between NuRD and chromatin. The PHD domains of CHD4 (and CHD3) bind nucleosomes (28, 59–61), and the PHD/chromodomains of CHD4 also regulate the CHD4 ATPase-helicase and chromatin remodeling activity (62, 63). It is, therefore, clear that the interactions of NuRD with nucleosomes are mediated by multivalent interactions (Fig. 9). An understanding of the interplay of these numerous NuRD-chromatin interactions will require structural studies of the intact NuRD complex.

## Footnotes

8 The abbreviations used are:

NuRD: nucleosome remodeling and deacetylase
NURF: nucleosome remodeling factor
PRC2: Polycomb repressive complex 2
ITC: isothermal titration calorimetry.
